# Effects of PARP-1 Deficiency on Th1 and Th2 Cell Differentiation

**DOI:** 10.1155/2013/375024

**Published:** 2013-11-05

**Authors:** M. Sambucci, F. Laudisi, F. Novelli, E. Bennici, M. M. Rosado, C. Pioli

**Affiliations:** ^1^ENEA, Laboratory of Radiation Biology and Biomedicine, Via Anguillarese 301, S. Maria di Galeria, 00123 Rome, Italy; ^2^Neuroimmunology Unit, Fondazione Santa Lucia, IRCCS, Via del Fosso di Fiorano 64, 00143 Rome, Italy; ^3^Singapore Immunology Network (SIgN), A∗STAR 8A Biomedical Grove, Singapore 138648

## Abstract

T cell differentiation to effector Th cells such as Th1 and Th2 requires the integration of multiple synergic and antagonist signals. Poly(ADP-ribosy)lation is a posttranslational modification of proteins catalyzed by Poly(ADP-ribose) polymerases (PARPs). Recently, many reports showed that PARP-1, the prototypical member of the PARP family, plays a role in immune/inflammatory responses. Consistently, its enzymatic inhibition confers protection in several models of immune-mediated diseases, mainly through an inhibitory effect on NF-*κ*B (and NFAT) activation. PARP-1 regulates cell functions in many types of immune cells, including dendritic cells, macrophages, and T and B lymphocytes. Our results show that PARP-1KO cells displayed a reduced ability to differentiate in Th2 cells. Under both nonskewing and Th2-polarizing conditions, naïve CD4 cells from PARP-1KO mice generated a reduced frequency of IL-4^+^ cells, produced less IL-5, and expressed GATA-3 at lower levels compared with cells from wild type mice. Conversely, PARP-1 deficiency did not substantially affect differentiation to Th1 cells. Indeed, the frequency of IFN-*γ* ^+^ cells as well as IFN-*γ* production, in nonskewing and Th1-polarizing conditions, was not affected by PARP-1 gene ablation. These findings demonstrate that PARP-1 plays a relevant role in Th2 cell differentiation and it might be a target to be exploited for the modulation of Th2-dependent immune-mediated diseases.

## 1. Introduction

CD4 T cells play central roles in the function of the immune system: They help B cells make antibody, enhance and maintain responses to CD8 T cells, regulate macrophage functions, orchestrate immune responses against a wide variety of pathogenic microorganisms, and regulate/suppress immune responses to prevent autoimmunity and to adjust the magnitude and persistence of responses. CD4 T cells are important mediators of immunological memory, and when their numbers are diminished or their functions are compromised, the organism becomes susceptible to a wide range of infectious diseases [1]. Naïve conventional CD4 T cells have open to them four (and maybe more others) different fates that are determined by the pattern of signals they receive during the initial interaction with antigen [2]. The classification is based on cytokine profiles and lineage-regulating transcription factors. T helper 1 (Th1) cells. considered as mediators of cellular immunity, are characterized by the transcription factor T-bet and expression of IFN-*γ*. By contrast, Th2 cells, mediators of humoral immunity, are distinguished by the transcription factor GATA-3 and a unique set of cytokines, including IL-4, IL-5, and IL-13 [3]. Naïve CD4 cells can also differentiate to Th17 cells, characterized by the production of IL-17 (and other) cytokines, in the presence of inflammatory signals, and to regulatory T cells, which maintain immune tolerance and modulate immune response amplitude, in the presence of a tolerogenic signals. Many studies showed that beside the leading cytokines, other factors are involved in T helper cell differentiation.

Recently, a growing body of evidence indicates that poly(ADP-ribose) polymerase 1 (PARP-1) plays a relevant role in inflammatory/immune responses. Poly(ADP-ribose) polymerases are enzymes that synthesize and bind branched polymers of ADP-ribose to acceptor proteins using NAD as substrate. PARP-1 accounts for the majority of the poly(ADP-ribose) polymer synthesis and functions as a DNA nick sensor and signaling molecule binding to single and double strand breaks [4]. However, PARP-1 is activated through several mechanisms in the absence of DNA damage. The relationship between PARP-1 activity and inflammation has been investigated for a long time with pharmacological PARP inhibitors and subsequently confirmed in PARP-1KO mice [5, 6]. The beneficial effects of PARP-1 inhibition in immune-mediated disease models result from a reduction in the expression of cytokines, chemokines, and other inflammatory mediators. Recent findings revealed that PARP-1 plays a role also in the adaptive immune response by modulating the ability of dendritic cells to stimulate T cells or by directly affecting functions of T and B cells [7–11]. We previously showed that PARP-1KO mice have greater numbers of regulatory T cells in thymus and peripheral lymphatic organs. PARP-1KO naïve CD4 T cells stimulated in the presence of TGF*β*1 generate higher percentages of Foxp3^+^ inducible regulatory T cells compared with WT cells, revealing that PARP-1KO affects T cell differentiation [12]. Poly(ADP) ribosylation has been also involved in Th2-mediated diseases. Following allergen challenge PARP-1 protein expression and activity are greatly increased in murine lungs, suggesting an involvement of this enzyme in allergic inflammation [13, 14]. Indeed, although IL-4 production was moderately affected in OVA-challenged, PARP-1^−/−^ mice, the production of IL-5, IL-10, IL-13, and GM-CSF was completely inhibited in ex vivo OVA-challenged lung cells derived from these animals [14]. Interestingly, a PARP-1 polymorphism which decreases PARP-1 activity was associated with a decreased risk of asthma in a Turkish population [15]. Considering the role of PARP-1 in inflammatory responses, this paper focuses on the effects of PARP-1 gene deletion on Th1/Th2 differentiation.

## 2. Materials and Methods

### 2.1. Animals

Female 10–12 weeks old C57Bl/6^WT^ and C57Bl/6^parp-1KO^ mice were used. All the experimental procedures were approved by the ethical committee of our institution and were performed according to the Italian laws and the European Union directives.

### 2.2. In Vitro Induction of Th1 and Th2 Cell Polarization

Naïve CD4^+^ (CD4^+^CD25^*‒*^CD62L^hi^) T cells were purified from spleens of 10–12-weeks-old mice by immunomagnetic cell sorting (CD4^+^ CD62L^+^ T cell isolation Kit II, 130-093-227; Miltenyi Biotec). Briefly, non CD4-expressing cells are labeled with a cocktail of biotin-conjugated antibodies against CD8*α*, CD45R, CD11b, CD25, CD49b, TCR*γ*/*δ*, and Ter-119 and antibiotin microbeads. The labeled cells are subsequently depleted by separation through LS columns (Miltenyi Biotec 130-042-401). In the following step, cells are directly labeled with anti-CD62L microbeads and isolated by positive selection from the preenriched CD4 T cell fraction through LS or MS columns (Miltenyi Biotec 130-042-201). Collected cells were found to be almost exclusively (>95%) CD4^+^CD45RB^hi^CD44^lo^ by flow cytometry analysis. To induce Th cell polarization, naïve CD4 T cells were cultured in 24-well plates (3524 Costar, Corning Inc., Corning, NY, USA) precoated with anti-CD3*ε* mAb (10 *μ*g/mL; clone 145-2C11, BD Pharmingen.). Anti-CD28 mAb in soluble form (1 *μ*g/mL; clone 37.51, BD Biosciences) or plate-bound recombinant CD80 (30 ng/mL; 740-B1, R&D Systems) or CD86 (30 ng/mL; 741-B2, R&D Systems) was added. In Th1-polarizing cultures, IL-12 (10 ng/mL; RMIL-12I, 419 ML, R&D System) and anti-IL-4 blocking mAb (10 *μ*g/mL; 11B11, BD Pharmingen) were also added. In Th2-polarizing cultures, IL-4 (5 ng/mL; RMIL-4I, Thermo Scientific, Rockford, IL, USA) and anti-IFN-*γ* blocking mAb (2.5 *μ*g/mL XMG1.2, BD Pharmingen) were added. CD4 T cells were also stimulated with anti-CD3 and CD28 mAbs in the presence of both anti-IFN-*γ* and anti-IL-4 blocking antibodies (neutral conditions). Cytokine-producing cells were identified by flow cytometry after restimulation with phorbol 12-myristate 13-acetate (PMA; 100 ng/mL) and ionomycin (1 *μ*g/mL) for 5 hrs. Brefeldin A was added after the first hour of restimulation.

### 2.3. Flow Cytometry Analyses

Cells were preincubated with anti-CD16/32 mAb (2.4G2; BD Pharmingen, San Diego, CA, USA) and then stained as previously described [16], with combinations of the following Abs: anti-CD4-biotin (H129.19), FITC-conjugated anti-IFN-*γ* mAb (clone XMG1.2), PE-conjugated anti-IL-4 mAb (clone BVD4-1D11) from BD-Pharmingen (San Diego, CA, USA), anti-GATA-3 rat IgG1 (clone HG3-31) from Santa Cruz Biotechnology Inc., and a FITC-conjugated anti-rat IgG (clone A85-1); and PE-conjugated anti-T-bet (clone 4B10) from eBioscience. Biotin-conjugated mAbs were revealed with avidin-PerCP (BD Pharmingen). For intracellular staining, cells were fixed and permeabilized using the Cytofix/Cytoperm Plus kit (555028; BD Biosciences, San Josè, CA, USA). Optimal concentrations of the antibodies and reagents were assessed in previous experiments. Fluorescence signals were collected in log mode using a FACSCalibur (Becton Dickinson) or a FACSCanto (Becton Dickinson). Analyses of cell populations were performed on events gated according to FSC/SSC parameters.

### 2.4. Cytokine Titration

IFN*γ*, IL-4, and IL-5 (KM-IFN*γ*, KM-IL4, and KM-IL5; Thermo Scientific, Rockford, IL, USA) were titrated in culture supernatants by sandwich ELISA according to the supplier protocols and as already described [16]. Absorbance was measured at 405 nm. The reference straight line obtained by plotting the absorbance versus the standard cytokine concentrations was used to calculate the cytokine concentrations in culture supernatants.

### 2.5. Transcript Analyses

Total RNA samples were extracted using a NucleoSpin RNA kit (MACHEREY-NAGEL). Reverse transcription was performed with reverse transcriptase, RNase inhibitor, random hexamers, and dNTP from EuroClone (EMR438250, EMR436250, EMR 428001, and EMR415501) according to the instructions from the supplier. cDNA amplification was performed using real-time fluorogenic 50-nuclease PCR with an ABI prism 7700 (Applied Biosystems) according to the manufacturer's instructions. Primers and probes for GAPDH, GATA3, and T-bet for cDNA amplification were from Applied Biosystems (assay IDs 4352339E; Mm00484683-m1; and Mm01299453_m1). Cycling conditions were set according to the manufacturer's instructions. The absence of PCR products from genomic DNA contamination and linearity between threshold cycle number and sample concentration were verified as previously described [12]. GATA3 and T-bet transcripts were normalized to GAPDH levels.

### 2.6. Statistical Analysis

A two-tailed Student's *t*-test for unpaired samples was used. Data represent mean values ± S.E. Significance levels are indicated in the figure legends. *P*-values < 0.05 were considered statistically significant.

## 3. Results

### 3.1. Reduced Th2 Cell Differentiation in PARP-1 KO CD4 Cells

**Figure 1:**
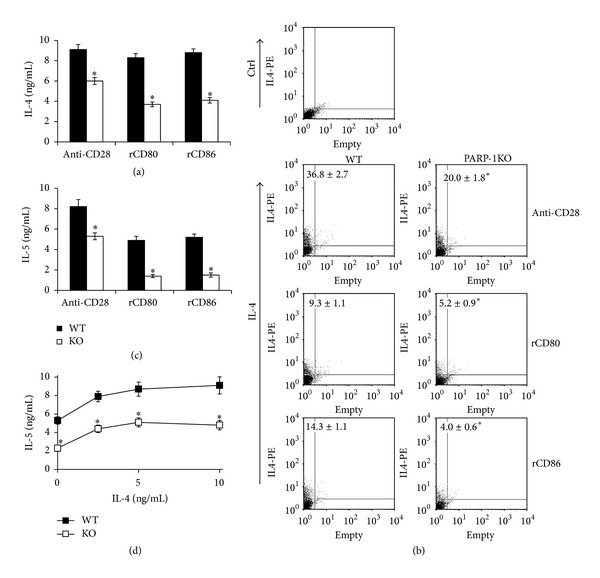
PARP-1 deficient cells display impaired Th2 cell polarization. Naïve CD4 T cells from wild type (WT, filled symbols) and PARP-1KO (KO, unfilled symbols) mice were stimulated with anti-CD3 mAb and either anti-CD28 mAb or recombinant CD80 or rCD86. (a) and (c) After 7 days of stimulation, culture supernatants were collected and analyzed by ELISA to assess IL-4 and IL-5 concentrations. (b) After 7 days of stimulation, cells were collected, restimulated with PMA and ionomycin for 6 hours in the presence of brefeldin A, and analyzed by flow cytometry to assess the frequency of IL-4-producing cells. Representative dot plots are shown. Numbers represent mean percentages of IL-4^+^ cells ± S.E.; ctrl, control antibody. (d) Culture supernatants from cells stimulated with anti-CD3 and rCD86 in the presence of graded concentrations of IL-4 (0–10 ng/mL) were analyzed for IL-5 by ELISA. Values represent means ± S.E. Results were confirmed in three other independent experiments. *P < 0.05 for KO versus WT cells in (a), (b), (c), and (d) panels.

Naïve CD4 T cells purified from wild type (WT) and PARP-1KO mice were stimulated under different culture conditions to evaluate Th2 cell differentiation. While the TCR/CD3 complex was stimulated with anti-CD3*ε* mAb, costimulatory signals were provided by stimulating the CD28 receptor either with an anti-CD28 mAb or with recombinant forms of B7 ligands, namely, CD80 (B7.1) or CD86 (B7.2). After 1 week of stimulation, PARP-1-KO cells produced less IL-4 than WT cells under all the stimulating conditions due to a reduced frequency of IL-4-producing cells (Figures 1(a) and 1(b)). Under the same conditions, also IL-5 production was reduced (Figure 1(c)). We wondered whether reduced Th2 cytokine production was also taking place when cells were stimulated under Th2-polarizing conditions. Cells were then stimulated with anti-CD3 mAb and rCD86 in the presence of anti-IFN*γ* neutralizing mAb and graded concentrations of IL-4. The results showed that PARP-1KO cells produced less IL-5 than WT cells also under these polarizing conditions, regardless of the IL-4 concentration (Figure 1(d)).

### 3.2. Reduced GATA-3 mRNA Expression in PARP-1 KO CD4 Cells

**Figure 2:**
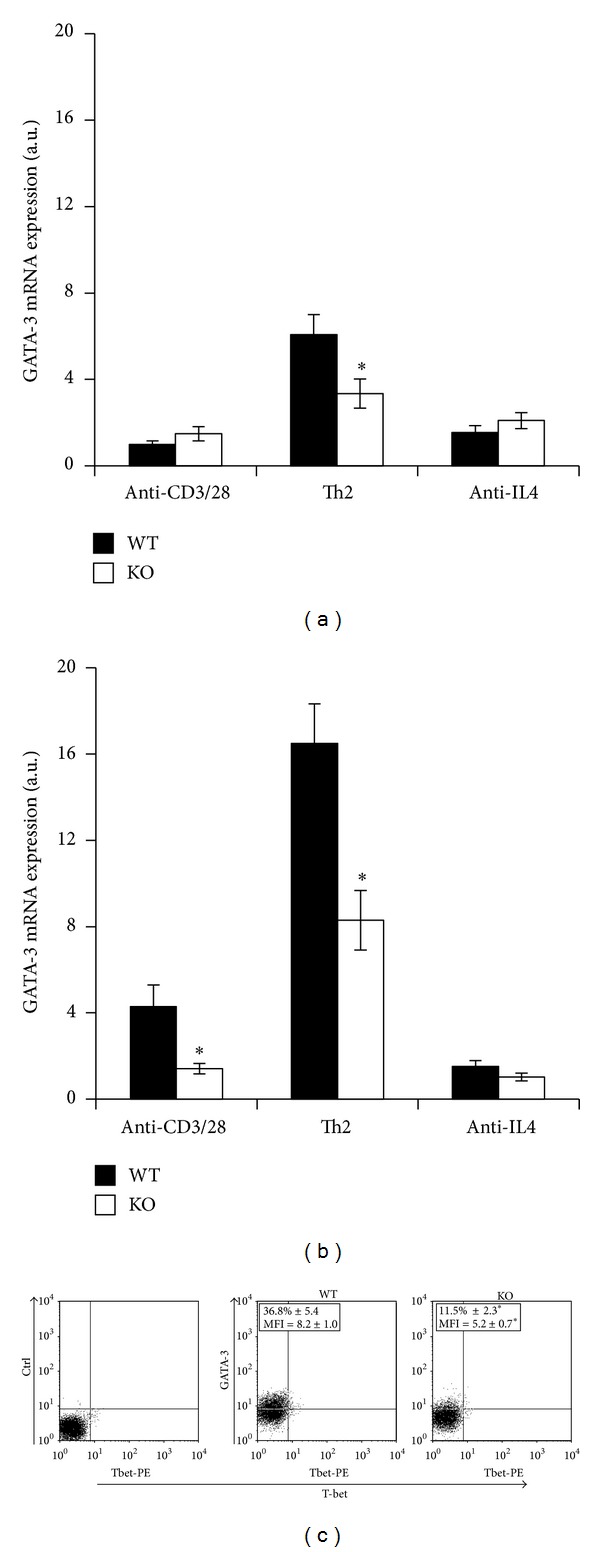
PARP-1 deficient cells express GATA-3 at lower levels than WT cells. Naïve CD4 T cells from wild type (WT, filled symbols) and PARP-1KO (KO, unfilled symbols) mice were stimulated with anti-CD3 and anti-CD28 mAbs alone or together with either IL-4 and neutralizing anti-IFN-*γ* mAb (Th2-polarzing conditions) or anti-IL4 neutralizing mAb (anti-IL4). GATA-3 mRNA expression was assessed by reverse transcription and real time PCR after 24 (a) and 72 (b) hours of stimulation. Results, normalized on the housekeeping gene (GAPDH) mRNA level, are referred to the GATA-3 mRNA level in WT cells stimulated for 24 hrs and expressed as arbitrary units. Values represent means ± S.E. from three independent experiments. (c) After 72 hours of stimulation with anti-CD3, rCD86 cells and IL-4 (and anti-IFN-*γ*) cells were analyzed by flow cytometry. Representative dot plots are shown. The indicated percentage of GATA-3-expressing cells and mean fluorescence intensity (MFI) for GATA-3 staining are means (±S.E.) from three independent experiments. *P < 0.05 for KO versus WT cells, where indicated in (a), (b), and (c).

Th2 cell differentiation is sustained by the transcriptional factor GATA-3, which exerts epigenetic and direct transcriptional effects on Th2 cytokine genes. To verify whether a reduced Th2 cell differentiation in PARP-1KO CD4 cells was depending on a reduced GATA-3 mRNA expression, naïve CD4 cells were stimulated under either neutral or Th2-polarizing conditions for 24 and 72 hrs. Upon CD3/CD28 costimulation, GATA-3 mRNA expression was readily upregulated and further boosted in the presence of IL-4 (Figure 2). Interestingly, PARP-1 KO cells expressed less GATA-3 mRNA compared with WT cells under both non- and Th2-polarizing conditions (Figures 2(a) and 2(b)). The addition of an anti-IL-4 antibody to cultures stimulated under nonpolarizing conditions reduced GATA-3 mRNA expression in WT cells, suggesting that the endogenous IL-4 plays a role in the difference observed in GATA-3 mRNA levels (Figure 2(b)). After 72 hours of stimulation under Th2-polarizing conditions, GATA-3 expression was also analyzed at protein level by flow cytometry (Figure 2(c)). Results showed that a higher percentage of naïve CD4 T cells from WT animals differentiated to GATA-3-expressing cells compared with PARP-1KO cells. Moreover, PARP-1KO cells expressed GATA-3 protein at lower level than that expressed by WT cells (MFI 8.0 versus 5.3).

Altogether, these findings showed that PARP-1KO naïve CD4 T cells have an impairment in Th2 cell differentiation depending on a reduced GATA-3 mRNA expression.

### 3.3. Effects of PARP-1 Deficiency on Th1 Cell Differentiation

**Figure 3:**
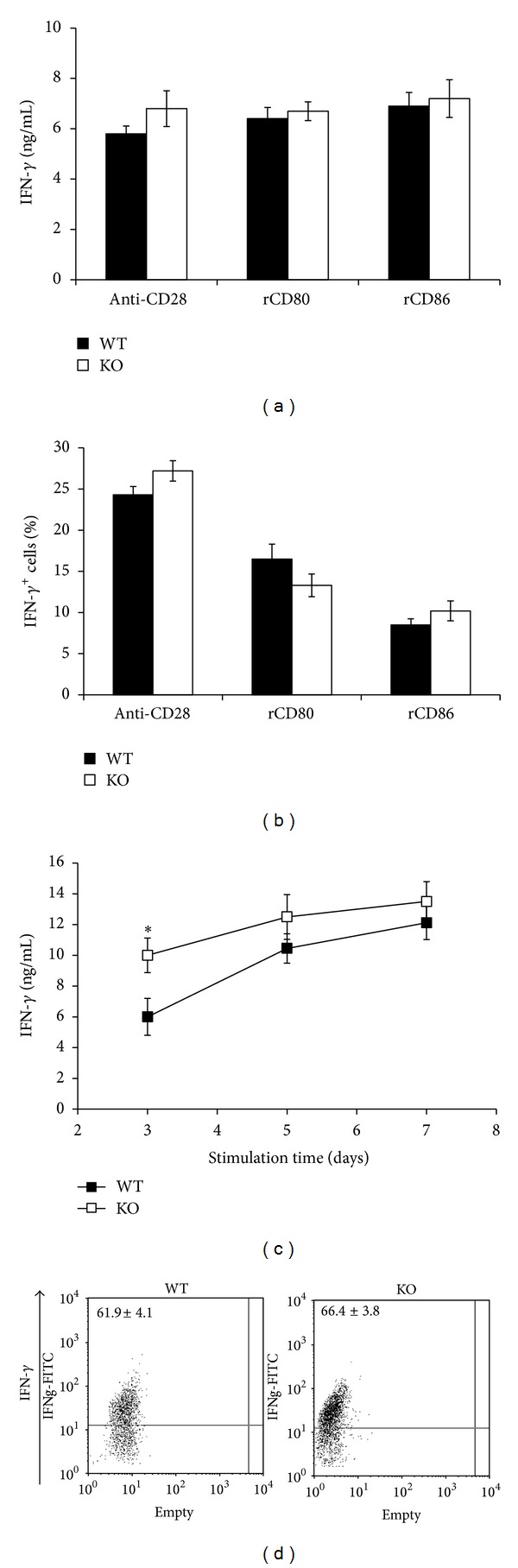
PARP-1 deficiency does not affect Th1 cell polarization. Naïve CD4 T cells from wild type (WT, filled symbols) and PARP-1KO (KO, unfilled symbols) mice were stimulated with anti-CD3 mAb and either anti-CD28 mAb or rCD80 or rCD86 for 7 days, ((a), (b), and (d)), or for the indicated time points (c). (a) and (c) IFN-*γ* concentration in culture supernatants as assessed by ELISA. (b) Cells were collected, restimulated with PMA and ionomycin for 6 hours in the presence of brefeldin A and analyzed by flow cytometry to assess the frequency of IFN-*γ*-producing cells. (c) IFN-*γ* concentration in cell cultures stimulated with anti-CD3, anti-CD28, IL-12, and neutralizing anti-IL-4 mAb (Th1-polarizing conditions). (d) Cells were stimulated under Th1-polarizing conditions for 7 days and, after restimulation as in (b), analyzed by flow cytometry. Values represent means ± S.E. *P < 0.05 for KO versus WT cells. Results were confirmed in three other independent experiments.

To assess whether PARP-1 controls the production of Th1 cytokines, naïve CD4 T cells purified from splenocytes of  WT and PARP-1KO mice were stimulated with anti-CD3 mAb and either rCD80 or rCD86 under nonskewing and Th1-polarizing conditions. Regardless of the costimulatory ligand used, WT and PARP-1 KO cells produced similar amounts of IFN-*γ* under nonskewing conditions (Figure 3(a)). Upon restimulation with PMA/ionomycin, cells were also analyzed for the expression of IFN-*γ* by flow cytometry. The results showed that in the presence of either B7 ligands, the percentages of IFN-*γ* ^+^ cells was not affected by PARP-1 deficiency (Figure 3(b)). The addition of IL-12 and a neutralizing anti-IL4 mAb (Th1-polarizing conditions) induced a higher IFN-*γ* production in PARP-1KO cells at early time points (Figure 3(c)). However, this difference decreased with the stimulation time, disappearing after 7 days. Flow cytometry analyses showed that a similar percentage of WT and PARP-1KO cells stimulated under Th1-polarizing conditions expressed IFN-*γ* (Figure 3(d)).

**Figure 4:**
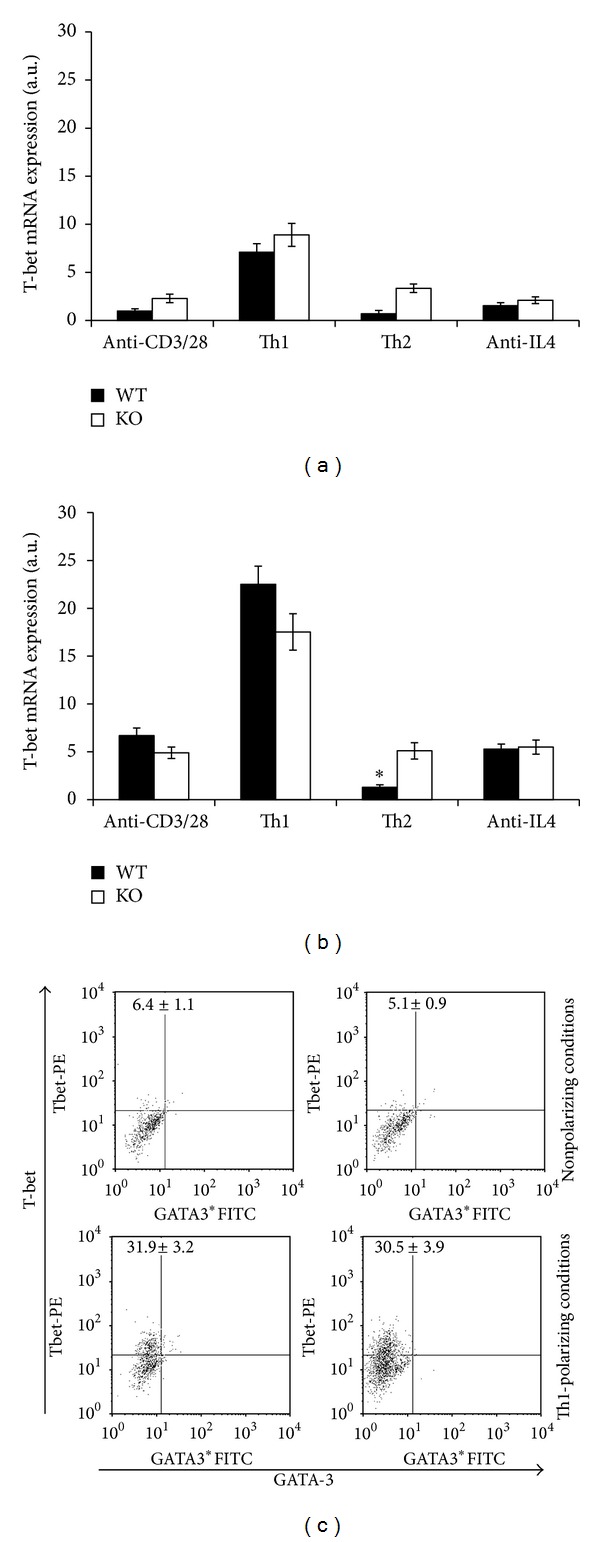
PARP-1 deficiency does not affect T-bet mRNA expression. (a) and (b) Naïve CD4 T cells from wild type (WT, filled symbols) and PARP-1KO (KO, unfilled symbols) mice were stimulated with anti-CD3 and anti-CD28 mAbs alone or together with either IL-12 and neutralizing anti-IL-4 mAb (Th1-polarizing conditions) or IL-4 and a neutralizing anti-IFN-*γ* mAb (Th2-polarizing conditions) or anti-IL4 neutralizing mAb (anti-IL4). T-bet mRNA expression was assessed by reverse transcription and real time PCR after 24 (a) and 72 (b) hours of stimulation. Results, normalized on the housekeeping gene (GAPDH) mRNA level, are referred to the T-bet mRNA level in WT cells stimulated for 24 hrs and expressed as arbitrary units. Values represent means ± S.E. Results were confirmed in two other independent experiments. (c) After 5 days stimulation with anti-CD3 and rCD86 alone (nonpolarizing conditions, upper panels) or together with IL-12 (and anti-IL-4) (Th1-polarizing conditions, lower panels), cells were analyzed by flow cytometry. Representative dot plots are shown. Numbers in dot plots represent means (±S.E.) from three independent experiments. *P < 0.05 for KO versus WT cells.

The IL-12/IFN-*γ* driven Th1 cell differentiation depends on the transcription factor T-bet. We analyzed the expression of T-bet at mRNA and protein levels. Naïve CD4 T cells were stimulated under either neutral or Th1-polarizing conditions for 24 and 72 hrs. After 24 hrs, Th1-polarizing conditions upregulated T-bet mRNA expression compared with CD3/CD28 costimulation alone, with the effect being higher after 72 hrs. Under these conditions, no differences between WT and PARP-1KO cells were found. Conversely, under Th2-polarizing conditions, T-bet mRNA expression was not upregulated in PARP-1KO cells and inhibited in WT cells compared with not-skewing conditions, with the effect being evident after 72 hrs of stimulation. T-bet expression was not altered by an anti-IL-4 mAb compared with nonpolarizing conditions (Figures 4(a) and 4(b)). After 5 days of stimulation, cells were analyzed for T-bet protein expression by flow cytometry. CD3/CD28 stimulation under non skewing conditions induced a small percentage of T-bet-expressing cells in both WT and PARP-1KO cell cultures (Figure 4(c), upper panels). Conversely, under Th1-polarizing conditions, almost one-third of the stimulated cells were expressing T-bet, with no differences between WT and PARP-1KO cells (Figure 4(c), lower panels). No GATA-3^+^ cells were observed under these conditions.

## 4. Discussion

Upon priming, naïve CD4 T cells undergo differentiation and acquire effector functions. TCR engagement, costimulation, and cytokines activate signalling pathways and transcription factors that synergize by promoting specific patterns of (cytokine) gene expression. Effector CD4 Th cells are divided in different cell populations according to the cytokine pattern expressed. Th1 and Th2 cells sustain distinct immune responses, which are protective for different groups of pathogens, through the production of IFN-*γ* and IL4/IL5/IL13, respectively. As both cell subsets are also involved in pathological processes, Th cell differentiation requires therefore the control of several factors to ensure that a protective not harmful immune response is developed.

Recent findings showed that PARP-1 plays an important role in the modulation of inflammatory/immune responses independently from DNA damage and NAD/ATP metabolism [17]. PARP-1 enzymatic inhibition affects dendritic cell maturation resulting in a reduced ability to stimulate T cell proliferation in a mixed leukocyte reaction [8]. PARP-1 inactivation has also T cell intrinsic effects as it controls the expression of several genes, IL-2 production, and T cell proliferation [11, 12].

Our results showed that upon TCR/CD28 costimulation, both GATA-3 and T-bet mRNA expressions are readily upregulated in naïve CD4 T cells. The addition of the polarizing cytokines IL-4 or IL-12 further boosted the expression of GATA-3 and T-bet, respectively. GATA-3 is a transcription factor necessary for Th2 cell differentiation. By allowing histone acetylation, GATA-3 induces changes in the chromatin structure of the *Il-4* locus that leads to derepression, acquisition of DNase hypersensitivity, and transcription of Th2 cytokine genes [18, 19]. At variance, epigenetic modifications in the *Ifn-*γ** gene are promoted by T-bet which plays a crucial role in Th1 cell differentiation [20, 21].

In contrast with previous results showing that in PARP-1KO T cells the expression of IFN-*γ* is increased [11], we found no substantial alterations in the differentiation of Th1 cells and T-bet expression. These findings obtained with a stimulating anti-CD28 mAb were also confirmed using the two natural ligands of CD28, namely, CD80 and CD86. Interestingly, cells from PARP-1KO mice revealed an impairment in Th2 cell differentiation with a reduced frequency of IL-4-producing cells and a reduced production of both IL-4 and IL-5. A reduction in Th2 cell differentiation was observed under both neutral and polarizing conditions. Moreover, neutralization of endogenous IL-4 decreased the difference between WT and KO cells, indicating that PARP-1KO cells seem to respond to IL-4 signalling in a different, less efficient, manner. We also found that PARP-1KO cells express GATA-3 at lower levels compared with WT cells. While, in the absence of polarizing cytokines, naïve CD4 T cells express GATA-3 at insufficient levels to promote full differentiation, upon IL-4 addition, GATA-3 expression is upregulated and its level becomes sufficient to sustain its own expression [22]. Our findings therefore suggest that in PARP-1KO cells, the positive feedback between IL-4 production and GATA-3 expression, which in WT cells reinforce the differentiation process, is partially compromised. Consistently, a recent report showed that lack or enzymatic inhibition of PARP-1 renders STAT6, which is required for IL-4 signaling, susceptible to calpain-mediated degradation, with consequent reduction in GATA-3 and IL-5 mRNA expression [23]. Interestingly, STAT6-dependent activity is regulated also by PARP-14. Upon IL-4 stimulation, PARP-14 ADP-ribosylates the histone deacetylases HDACs present at the Gata3 promoter favoring STAT6 binding [24, 25]. Thus, GATA-3 mRNA expression and therefore Th2 cell differentiation are regulated at different levels by multiple members of the PARP family.

Our previous findings [12] showed that PARP-1 negatively regulates naïve CD4 cell differentiation to regulatory T cells without affecting the TGF*β*1/IL-6 driven differentiation to Th17 cells. Altogether, these findings suggest that PARP-1 might be a target to be exploited for the modulation of Th2-dependent immune-mediated diseases.
